# Physician related barriers towards insulin therapy at primary care centres in Trinidad: a cross-sectional study

**DOI:** 10.1186/s12875-020-01271-1

**Published:** 2020-09-21

**Authors:** Shastri Motilal

**Affiliations:** Department of Paraclinical Sciences, Faculty of Medical Sciences, University of the West Indies, St. Augustine Campus, Eric Williams Medical Sciences Complex, Champs Fleurs, Trinidad

**Keywords:** Insulin, Insulin barriers, Type 2 diabetes mellitus, Primary care

## Abstract

**Background:**

Physician related factors with respect to insulin therapy can contribute to diabetes mellitus (DM) mismanagement. Patient related factors have been previously explored in a Trinidad survey. The main objective of this study was to explore primary care physicians’ (PCPs) related barriers towards insulin therapy.

**Methods:**

A cross-sectional study on a convenience sample of PCPs in the public primary care system was done using an online survey.

**Results:**

Of the 170 PCPs contacted, 75 (44%) responded. There were 47 females (62.7%) and 28 males (37.3%) with a mean age of 35.9 yrs. Nearly 40% of physicians admitted that the education given to patients was inadequate to allow initiation of insulin therapy. Half the respondents admitted to insufficient consultation times and inadequate appointment frequency to allow for intensification of insulin therapy. Forty percent of PCPs admitted that HbA1c results were unavailable to guide their management decisions. Only 6.7% of physicians said they had access to rapid acting insulin, while 5.3% said they had access to insulin pens.

**Conclusion:**

PCPs in Trinidad treating diabetes at the public primary care clinics face several barriers in administering proper insulin therapy. Addressing these factors can improve glycemic control in this population.

## Background

Trinidad and Tobago are a twin island state in the Southern Caribbean with Trinidad accounting for 95% of the population. Occupying just over 5128 km^2^, Trinidad and Tobago has a high prevalence of diabetes mellitus (DM) with 12.3% of the adult population affected according to the International Diabetes Federation [1]. In 2016 DM accounted for 14% of all deaths and its economic burden in the region have been well documented [2, 3]. Poorly controlled DM is a major contributor to morbidity and mortality. The Caribbean Health Research Council (CHRC) guidelines, endorsed by the Caribbean College of Family Physicians, supports an HbA1c of less than 6.5% as the target for glycemic control [4]. Previous Trinidadian studies have shown 40–55% of DM patients had an HbA1c above this recommended target [5, 6].

Diabetes is a progressive disease that is initially managed with lifestyle change, metformin and other oral hypoglycemic therapies. Insulin treatment is the major definitive effective treatment for those who remain uncontrolled on oral treatments [7]. Insulin has been available free of charge to Trinidad citizens since 2003 under the CDAP (chronic disease assistance programme) with satisfaction in the majority of CDAP drug users [8]. While the reasons for uncontrolled DM are complex and multifactorial, one study found that insulin refusal, lack of insulin titration, lack of knowledge of diabetes, side effects and infrequent clinic attendance were some of more common contributors to uncontrolled DM [9].

Inertia to instituting and intensifying insulin therapy has been a well-documented phenomenon, with both patient and clinician related factors [10, 11]. Clinician barriers have also been described in both primary care providers (PCPs) and specialists [12–14]. A previous Trinidadian survey explored DM patients’ knowledge, attitudes and perceptions towards insulin treatments [15]. The primary objective of this study was to investigate the barriers that the PCPs of these patients face, regarding insulin therapy. The secondary objective was to compare PCP barriers with that of the patients from the previous study done.

## Method

### Setting and sampling strategy

This was a cross-sectional study done by an online survey of PCPs in the public health system of Trinidad. There exists a two-tiered system of public and privatized care on the island with public health care delivered through 4 Regional Health Authorities (RHAs) in TT. All the physicians who were actively employed under the RHAs formed the target population of PCPs for this study. Following approvals from the Ethics Committee of the University of the West Indies, St. Augustine Campus, and each health authority, a listing of all PCPs was sought from all RHAs. They were contacted by email and invited to participate in the online survey following online informed consent during the period April – June 2013. Up to 4 email reminders were sent to non-responding physicians and there were no incentives offered for participation.

### Sample size

The physician population was limited to the total number of PCPs in the Trinidadian public health system at the time of the survey which was 170.

### Development of instrument

A questionnaire was designed de novo based on literature searches for common barriers to insulin use. The PubMed (National Library of Medicine) and Caribbean Search (EBSCO) databases were searched [16, 17]. Keywords used were insulin therapy, initiation, intensification, barriers, primary care, general practice, and outpatient. Several papers which looked at insulin therapy in the outpatient or primary care setting were identified [18–20]. Common themes regarding insulin therapy were extracted in the areas of physician perceived patient barriers, technical administration, support from other health care professionals, consultation duration and frequency, and insulin types and devices. The first section of the survey focused on the barriers towards initiating insulin therapy and the second focused barriers towards insulin intensification. Demographics and data on insulin types and delivery systems were also included. The tool was pilot tested online with 10 PCPs and minor revisions and additions were made which were felt relevant to the local setting. The PCPs that participated in the pilot testing were also included in the group (170) that received the final survey.

### Analysis strategy

SPSS version 17 was used to analyze the data with categorical variables reported using frequencies and proportions. Chi squared and Fishers exact tests were used to compare independent groups of categorical variables. Planned comparisons were also made between PCP barriers found in this study with that of the previous Trinidadian patient survey.

## Results

### Demographics

The physician survey was sent to 170 primary care physicians, 75 of whom responded (44%). There were 47 females (62.7%) and 28 males (37.3%). The mean age of the physicians was 35.9 (SD = 8.2, range = 25–62) The sample comprised of PCPs from all four RHAs of which 10 were from the Eastern RHA (13.3%), 26 from the North Central RHA (34.7%), 14 from the North West RHA (18.7%), and 25 from the South West RHA (33.3%). The ethnicity of physicians included East Indian (66.7%), African (19.4%), mixed (9.7%), Caucasian (2.8%) and other minority races (1.4%). Physicians saw an average of 42 diabetic patients per week and were practicing for an average of 6 years.

### Insulin and delivering devices

When asked about the type of insulin devices their patients generally had access to, 100% of PCPs had access to syringes (insulin needles). On the other hand, only 5.4% of PCPs had access to pens and none reported access to insulin pumps.

In terms of the types of insulin available to PCPs, all physicians (100%) had 70/30 mixed insulin and 98.7% had long acting insulin (insulin glargine). The rapid acting insulin was the least widely available with only five (6.7%) of the PCPs offering that form of insulin.

### PCP barriers towards initiating insulin therapy

PCPs were asked to select criteria for initiation of insulin therapy. The most popular criteria selected were the degree of hyperglycemia/HbA1C levels (97.1% of physicians), if the patient was on maximum doses of oral hypoglycemic agents with an HbA1C > 7% (94.4%) and whether the patient was willing to try it (73.2%). Other criteria included: the patient’s work schedule and lifestyle factors (50.7%), how relevant the potential side effects of insulin are to the patient compared with those of other hypoglycemic agents (40.8%), the availability of nurses, diabetes educators and others to implement and follow the insulin treatment (39.4%), if the fasting plasma glucose is more than 250 mg/dl (35.2%) and the cost of insulin (11.4%).

PCPs were also asked about patient-perceived barriers towards initiating insulin therapy which are shown in Table 1. The most popular barriers were fear of needles (98.6%), lack of education (84.5%) and technical difficulty involved in administration (78.6%). Other factors which PCPs felt were barriers to patients initiating insulin therapy were inadequate support from family or caregivers (69%), attacks of hypoglycemia (43.7%), embarrassment/social stigma (23.9%), fear of death (22.5%), fear of weight gain (20%), inadequate support from nursing staff (15.5%), inadequate support from specialists (14.1%), religious/cultural beliefs (9.9%), decreased life span (8.6%) and financial issues (8.5%).

**Table 1:** Summary of the primary care physician perceived barriers with regard to initiating insulin therapy

Barrier towards initiating therapy (***n*** = 75)	%
Patient fear of needles	98.6%
Lack of education	84.5%
Technical difficulty involved in administration	78.6%
Lack of support from family or caregivers	69.0%
Attacks of hypoglycemia	43.7%
Embarrassment/social stigma	23.9%
Fear of death	22.5%
Fear of weight gain	20.0%
Inadequate support from nursing staff	15.5%
Inadequate support from specialists	14.1%
Religious/cultural beliefs	9.9%
Reduced life span	8.6%
Financial issues	8.5%

Of the respondents, 37% felt the education given to patients was not adequate to allow insulin initiation. Furthermore, more than half (53.5%) of PCPs felt that the consultation time was not sufficient to initiate insulin therapy while 50.7% felt that appointment frequencies was not sufficient to allow for review of a patient starting insulin therapy. In addition, 32.4% of physicians admitted that HbA1C results were not readily available to guide their decisions to initiate insulin therapy and 9.7% of physicians admitted that they were not confident in initiating insulin therapy on their own.

Comparisons were made between key PCP perceived barriers and that of their patients as reported in the aforementioned local patient survey [15]. Table 2 shows these comparisons. As seen in Table 2 there were no significant differences between PCP perceived patient related barriers and that of non-user diabetics towards commencing insulin therapy. Both groups had similar perceptions with regards to the barriers of weight gain, hypoglycemic spells, reduced lifespan, and embarrassment.

**Table 2:** Comparisons of perceptions of non- users with that of Primary Care Physicians with regard to key barriers in initiating insulin therapy

Barrier	Physician’s Perception of barriers facing non-users	Non-users Perception	***P*** values
Insulin causes weight gain	20% (15/75)	23.1% (63/275)	0.592
Insulin leads to a Shorter Life	8.6% (6/70)	6.4% (26/275)	0.820
Insulin leads to attacks of Low Blood Sugar	43.7% (33/75)	40.7% (111/275)	0.571
Embarrassment about taking insulin	23.9% (18/75)	28.2% (77/275)	0.490

### PCP barriers towards continuing or intensifying insulin therapy

The most common considerations PCP selected for insulin continuation or intensification were the degree of hyperglycemia/HbA1c levels (95.8%), patient compliance with existing regimen (80.3%), the risk of hypoglycemia (45.1%) and whether the patient was willing to increase insulin doses (36.6%). Other factors included the patient’s work schedule and lifestyle factors (22.5%), the availability of nurses, diabetes educators and others to follow the insulin treatment (12.7%) and the cost of insulin (4.2%).

Physicians were also asked about the barriers that they perceive their patients faced towards continuing/intensifying insulin therapy which are shown in Table 3. The most common perceived patient barriers among PCPs were hypoglycemia attacks (76.4%), inadequate support from family or caregivers (63.9%), frequency of administration (62.5%), fear of needles (50%) and technical difficulty involved in administration (40.3%). Additional perceived barriers included fear of weight gain (27.8%), inadequate support from nursing staff (12.5%), embarrassment/social stigma (6.9%), decreased life span (4.2%) and inadequate specialist support (4.2%).

**Table 3:** Summary of the primary care physician perceived barriers regarding intensifying insulin therapy

Barrier towards intensifying therapy (***n*** = 75)	%
Attacks of hypoglycemia	76.40%
Lack of support from family or caregivers	63.90%
Frequency of administration	62.50%
Patient fear of needles	50.00%
Technical difficulty involved in administration	40.30%
Fear of weight gain	27.80%
Inadequate support from nursing staff	12.50%
Embarrassment/social stigma	6.90%
Inadequate support from specialists	4.20%

When asked if their insulin dependent patients complained about the frequency of insulin administration, 54.9% of physicians said *Yes*. Half of PCPs stated they felt they did not have adequate consultation times and 51.4% of physicians admitted that appointment frequencies were not sufficient to allow review of patients who required intensification of their insulin regimen.

In addition, 40% of physicians admitted that HbA1c results were not readily available to guide their decision to intensify insulin therapy.

Gender, age, RHA, ethnicity, number of years in practice and number of patients seen weekly were however not predictive of physician responses to appointment times and HbA1c result availability. (*P* > 0.05).

## Discussion

By examining the barriers that PCPs face in administering insulin therapy, this study was able to identify several factors in the Trinidadian public primary care system that could explain why Trinidadian diabetics are not meeting glycemic targets. These include, need for education of patients, a failure to conduct regular assessment of HbA1c, inadequate time for consultations, infrequent appointments, limited access to rapid acting insulins and lack of insulin delivery devices.

Based on the results gathered nearly 40% of physicians admitted that the education given to patients was inadequate to allow initiation of insulin therapy. Nearly 10% of PCPs admitted that they were not confident enough to initiate insulin therapy on their own. These results indicate the need for physician and patient education regarding insulin therapy. Other similar surveys have highlighted gaps in physician education and recommended targeted medical education programs to improve physician knowledge, attitudes and beliefs [21–23]. In this study PCPs also displayed several perceived patient barriers which mirrored that of the non-users with regards to insulin initiation in the Trinidadian patient survey [15]. Both groups agreed that weight gain, hypoglycemia, and embarrassment about having to take insulin were barriers to its use. This represents areas for education for patient and PCP alike if insulin is to be instituted when indicated.

Recent HbA1c result unavailability was another physician barrier, independent of PCP demographics, RHA and number of patients seen. PCPs are unable to make important decisions regarding insulin initiation or intensification in the absence of such a basic clinical benchmark. This seems to mirror practice as from the local patient survey, recent HbA1c results could only be found in less than half of the patients’ charts [15]. Improving the capacity of the existing public laboratory services is needed. Point-of-care testing also has a vital role in increasing access to HbA1c testing. It has been shown to be a cost effective and impactful testing strategy in reducing clinical inertia and improving glycemic control [24–26].

Appointment times and consultation frequency were other barriers that PCPs cited in this survey. Roughly half of PCP admitted that they did not have enough consultation time. On average each PCP saw over 40 DM patients each week. The Trinidadian public primary care system is enacted through a network of 87 centres (78 Health Centres and 9 District Health Facilities). The majority (78%) of these centres offer “Diabetes Clinic” or “Chronic Disease Clinic” on only 1 day of the week [27]. This means most of the PCPs in this survey saw 40 diabetic patients on one typical 7-h workday or had 10 min allotted on average per patient. Is this enough time to manage the diabetic patient requiring insulin therapy and to address other targets? The inadequacy of time for proper chronic disease management and its negative impact on diabetes care in the primary care setting has been highlighted by others [28, 29]. A negative impact on diabetes care with larger panel size in a family medicine setting has also been demonstrated [30]. Alternative methods in primary care service delivery as well as training to make PCPs more systematic and efficient in their approach have been suggested as possible solutions [28, 29]. A local study highlighted the benefit of a dedicated PCP and registered nurse in improving diabetes care at a primary care center [31]. Through longer than average consultation times and more frequent appointments, the author was able to produce sustained reductions in Hba1c for up to 3 years [31]. The prevalence of DM in the Trinidad population of 120,000 means a DM patient to PCP ratio upwards of 700:1 in the public health system [1]. While there is no universally agreed upon optimal primary care panel size, this statistic can be considered in planning human resource needs for PCPs and other staff members with PCP supporting roles for enhancing diabetes management.

One third of the DM patients at the public clinics were on insulin [15]. The insulin preparations available at the time of this study in the public health system Trinidadian formulary were Insulin Glargine, Insulin Human Isophane 70/30, Insulin Human NPH and Insulin Human Regular. These were also the same preparations available at the time of the last formulary update posted [32]. These preparations are available free of charge to all citizens through the health center dispensaries. CDAP also provides free access to Regular, NPH and 70/30 insulins [8]. While provision of these insulins has undoubtedly contributed to glycemic control for patients, only 7% of PCPs in this survey had access to rapid acting insulin for their patients. Rapid acting insulin, however, is not on formulary and is only available for purchase in the private sector. Systematic reviews have demonstrated the superiority of rapid acting insulin over Regular insulin as it produces less nocturnal hypoglycemia, better glycemic control and improved patient satisfaction [33–35]. Insulin pens were also unavailable for most PCPs in this survey. Insulin pens have been associated with improved adherence and better self-management while remaining cost-effective [36–38]. Consideration should be given to expanding the existing Trinidadian public formulary to include rapid acting insulin and insulin pen devices for the benefits presented. Lastly insulin pumps were not an available option in this survey for PCPs. The American Diabetic Association states that pumps may be considered as an option for patients with type 1 diabetes who are able to safely manage the device [39]. The decision to use pumps in this subgroup of diabetics should be individualized after consultation with a specialist familiar with its use. For this reason, pumps should not be made routinely available for PCPs granted they are provided with the other insulin options as discussed above.

This study was the first to survey PCPs within the Trinidadian public health system, exploring common barriers to insulin use. PCPs were selected from all four RHA’s providing a good representation across Trinidad. The online nature of the survey also reduced both time and cost in the acquisition of physician responses. The main limitation of this study however was the low physician response rate (44%) This could have resulted in a biased estimation of the characteristics of this group. Such a convenient sample limits the generalizability of this study’s findings. Due to costs, no incentives were offered to improve response. This response rate, however, fell within the range of 20–47% which was highlighted in an article that compared response rates of online and paper-based surveys [40]. Furthermore no PCPs were surveyed in the less populous isle of Tobago which is run by a separate RHA. A Barbadian study that compared physician perception towards insulin use highlighted key differences between public and private PCPs [23]. In this regional study significantly more privately hired PCPs thought that the healthcare system allowed enough flexibility of time for education and initiating insulin was easy [23]. This survey did not approach private sector PCPs who may have had experienced different barriers compared to those in the public system.

## Conclusion

In conclusion this survey of PCPs highlighted education, limited HbA1c results, inadequate consultation time, infrequent appointments, limited access to rapid acting insulins and lack of insulin pens, as challenges in the effective management of DM in the Trinidad public health sector. It is important that these issues be addressed through various interventions including PCP education, improved HbA1c access, and investment in alternative forms of insulin and delivery devices. This will improve the initiation/intensification of insulin therapy in a timely manner resulting in less DM complications. Further research should focus on Trinidad’s private health sector and Tobago to investigate potential barriers, which may have implications for interventions specific to these settings.

## Supplementary information

**Additional file 1.**

## Data Availability

The datasets during and/or analyzed during the current study available from the corresponding author on reasonable request.

## Abbreviations

CDAP: Chronic Disease Assistance Programme (A program where all citizens can obtain basic chronic disease medications free of charge)
CHRC: Caribbean Health Research Council (A recognized body that fosters Caribbean research and best practice, now incorporated under the wider group; Caribbean Public Health Agency)
DM: Diabetes mellitus
PCP: Primary Care Physicians
RHA: Regional Health Authority
