# Supplementary material for: Physician related barriers towards insulin therapy at primary care centres in Trinidad: a cross-sectional study
Source: BMC Fam Pract. 2020 Sep 21;21:197. doi: 10.1186/s12875-020-01271-1 (PMC7507810; doi:10.1186/s12875-020-01271-1)
Supplement: Supplementary file 1 — Additional file 1. [file 12875_2020_1271_MOESM1_ESM.docx]

PHYSICIAN QUESTIONNAIRE

According to the National Diabetes Association of Trinidad and Tobago, there are as many as 175,000 persons with diabetes in Trinidad and Tobago. Although insulin is one of the primary treatments, many patients and physicians admit to indifference towards insulin therapy. Studies have been conducted on the knowledge, attitudes and perceptions of patients and primary care physicians in many countries including the US, UK and South Africa. However, limited studies have been conducted both nationally and regionally. This project seeks to investigate the aforementioned topic in Trinidad. It would be greatly appreciated if you completed this brief online questionnaire to aid in this investigation.

The questionnaire will be completed in two main parts:

1. Knowledge, Attitudes and Perceptions (KAP) of Physicians towards Initiating Insulin Therapy
2. Knowledge, Attitudes and Perceptions (KAP) of Physicians towards Continuing /Intensifying Insulin Therapy

**Demographic/Contact Information:**

1. Email address: ___________________________________
2. Specify your age? _____
3. What is your gender?
   - Male
   - Female
4. Specify the highest level of education attained.
   - MBBS
   - MD
   - Diploma
   - MsC
   - DM
   - PhD
   - Fellowship
   - Other
5. What is your ethnicity:
   - - African
     - East Indian
     - Other
6. Name and Address of Health Center where you treat patients:

_____________________________________________________________________

_____________________________________________________________________

____________________________________________________________

1. Which RHA does this health center belong to?
   - North Central (NCRHA)
   - North West (NWRHA)
   - South West (SWRHA)
   - East (ERHA)
2. How many diabetic patients do you generally treat per week? ______
3. Of this number, roughly what percentage is on insulin therapy?
   - 0-25%
   - 26-50%
   - 51-75%
   - 76- 100%
4. What types of insulin are accessible to you for prescription to patients? (You may specify more than one.)
   - 70/30 Insulin (Mixed)
   - Rapid acting insulin (Insulin glulisine or insulin aspart)
   - Short Acting Insulin (Insulin R)
   - Intermediate Acting Insulin (Insulin N)
   - Long Acting Insulin (Insulin Lantus)
5. What types of insulin devices are generally accessible to your patients? (You may specify more than one.)
   - Insulin Needles/Syringes
   - Pens
   - Insulin Pumps

**Part I: KAP of Physicians Towards Initiating Insulin Therapy**

1. Are you confident in initiating insulin therapy on your own?
   - Yes
   - No
2. Which of the following (if any) do you consider as criteria for initiating insulin therapy? [You may chose more than one]

o Whether the patient is willing to try it

o The degree of hyperglycemia (HbA1c level) o Fasting plasma glucose > 250 mg/dl

1. How relevant the potential side effects of insulin are to the patient compared with those of other hypoglycemic agents
   1. Patient is on maximum doses of oral hypoglycemic agents with HbA1c > 7%
   2. The patient‟s work schedule and lifestyle factors
   3. Cost of insulin
   4. The availability of nurses, diabetes educators, and others to implement and follow the insulin treatment.
2. In your opinion, what are some is the biggest barrier(s) patients face in initiating insulin therapy? (You may select more than one.)
   1. Fear of needles

o Fear of weight gain o Lack of education

o Attacks of low blood sugar (hypoglycemia) o Technical difficulty in administration

o Embarrassment/Social Stigma o Fear of death

o Financial issues

o Decreased Life Span

o Religious/Cultural Beliefs

o Other ____________________

1. Do you think the education given to the patient is adequate to allow insulin initiation?
   - Yes
   - No
2. Are HbA1c results readily available to guide your decision to initiate insulin therapy?
   - Yes
   - No
3. Do you think you have enough consultation time to initiate insulin therapy?
   - Yes
   - No
4. Are appointment times sufficient to allow for review of a patient starting insulin therapy?
   - Yes
   - No

**Part II: KAP of Physicians Towards Continuing/Intensifying Insulin Therapy**

1. Which of the following (if any) do you consider as criteria for intensifying insulin therapy? [You may chose more than one]

o Whether the patient is willing to increase insulin doses o Risk of hypoglycemia

o The degree of hyperglycemia (HbA1c level)

o The patient‟s work schedule and lifestyle factors o Cost of insulin

o The availability of nurses, diabetes educators, and others to follow the insulin treatment. o Patient compliance with existing regimen

1. Do your insulin dependent patients complain about frequency of administration?
   - Yes
   - No
2. If yes, would you decrease the frequency or switch to another method of treatment?
   - Decrease Frequency
   - Switch to another method of treatment
3. Are HbA1c results readily available in order to guide your decision to intensify insulin therapy?
   - Yes
   - No
4. Do you think you have enough consultation time with the patient in an attempt to intensify insulin therapy?
   - Yes
   - No
5. Are appointment times sufficient to allow review of patients who are intensifying their insulin therapy?
   - Yes
   - No
6. How would rate patient compliance towards insulin therapy?
   - - Poor
     - Fair
     - Good
7. In your opinion, what are the biggest barriers for your patients towards continuing/intensifying insulin therapy? (you may choose more than one):

o Fear of needles

o Fear of Weight Gain

o Decreased Life Span o Hypoglycemia Attacks

o Technical difficulty involved in administration o Frequency of administration

o Social stigma/ Embarrassment
